# Obesogenic Environment in the medical field: First year findings from a five-year cohort study

**DOI:** 10.12688/f1000research.125203.1

**Published:** 2023-05-25

**Authors:** Jo Ann Andoy-Galvan, Shyamkumar Sriram, Tey Jin Kiat, Lim Zig Xin, Wong Jun Shin, Karuthan Chinna

**Affiliations:** 1 School of Medicine, Faculty of Health and Medical Sciences, Taylor's University, Subang Jaya, Selangor, 47500, Malaysia; 2 School of Health Sciences and Professions, Department of Social and Public Health, Ohio University, Athens, OH, 45701, USA; 3 Faculty of Business and Management, UCSI University, Cheras, Kuala Lumpur, 56100, Malaysia

**Keywords:** Cohort, obesity, overweight, medical students

## Abstract

**Background:** Doctors with a normal BMI and healthy living habits have shown to be more confident and effective in providing realistic guidance and obesity management to their patients. This study investigated obesogenic tendencies of medical students as they progress in their medical studies.

**Methods:** A cohort of forty-nine medical students enrolled in a five-year cohort study and was followed up after one year. At the initiation of the cohort, socio-demography and information on anthropometry, accommodation, eating behavior, stress and sleeping habits of the students had been recorded. Follow-up data was collected using a standardized self-administered questionnaire.

**Results:** Thirty-seven percent of the students in the cohort are either obese or overweight in the one-year period.. A year of follow-up suggests that there is an increase in BMI among the male students (P=0.008) and the changes are associated with changes in accommodation (P=0.016), stress levels (P=0.021), and sleeping habits (P=0.011).

**Conclusion:** Medical education system should seriously consider evaluating this aspect in the curriculum development to help our future medical practitioners practice a healthy lifestyle and be the initiator of change in the worsening prevalence of obesity worldwide.

## Introduction

It is widely recognized that obesity is influenced by behavioral and genetic factors. However, recently, environmental factors are being investigated as it can be an independent risk factor in the causation of obesity which the medical community calls the “obesogenic environment”. ^1^ The term ‘obesogenic environment’ has been defined as ‘an environment that promotes weight gain and not conducive to weight loss,’ which is believed to be the driving force of the escalating obesity epidemic. ^2^

According to the World Health Organization (2020), obesity is defined as excessive or abnormal fat accumulation in the body and it is measured by Body Mass Index (BMI). Obesity increases the risk to health through developing various non-communicable diseases such as diabetes, stroke, and cardiovascular diseases. ^3^ The worldwide prevalence of obesity has tripled from 1975 to 2016, resulting in a global obesity epidemic by reckoning over 650 million adults with obesity in 2016, and an estimated one billion adults with obesity by 2025. ^3^^,^^4^ Recent investigations have evolved from imbalance of energy intake and consumption to establishing “obesogenic environment”. These environmental factors include the availability of high-calorie, low-nutrient foods, larger portion sizes, and an increase in sedentary activities due to technological advancements. ^5^ The obesity prevalence presented by WHO will be higher if the Asian BMI cut-off value (≥27.5 kg/m ^2^) is used instead of WHO international classification (≥30 kg/m). ^6^ In Malaysia, according to the National Health and Morbidity Surveys (NHMS), the prevalence of obesity in adults aged 18 years and above was 29.1% in 2006 and 33.7% in 2019, an increase of 15.8% in a decade now. Malaysia is the most obese Southeast Asian country. ^7^^–^^9^

Nobody is immune to obesogenic tendencies, including doctors and medical students. ^10^ Studies show that medical students are actually more prone to obesity and weight gain due to the nature of their curriculum which leads to  lack of leisure time, sedentary lifestyle, and increased stress as they progress towards clinical year. ^11^^,^^12^ Doctors with a normal BMI and healthy living habits have shown to be more confident and effective in providing realistic guidance and obesity management to their patients. ^13^^,^^14^ A study conducted at a Malaysian medical university found that the prevalence of obesity and overweight among the medical students was high at 30%. ^15^

A study conducted in 2019 in the same university revealed low prevalence of obesity of medical students in comparison with the national prevalence of the same age group though male students were found out to be 4.3× more likely to be obese compared to females. This study recommends further exploration of obesity development among medical students as they progress in their studies as there is no existing available data on this. ^16^ Therefore, this prospective study is conducted to determine the obesogenic tendencies of male and female medical students as they progress in their medical training and identify the risk factors involved. This paper presents the first year findings of the said prospective study.

## Methods

### Study design and setting

A prospective cohort study with yearly follow-up was initiated in 2019 among first-year medical students. In the cohort, socio-demography, measurement of height and weight, details about their accommodation, eating behavior, stress, and sleeping habits had been recorded by data collectors as Semester 1. Subsequent follow-up data were collected using a standardized self-administered questionnaire six months after as Semester 2 and one year after as Semester 3.

### Participants

Our study targeted all first-year medical students presently enrolled during the study period.

### Questionnaire

The questionnaire used in this research was designed with reference to several studies conducted by different universities. ^11^^,^^12^^,^^15^^,^^17^ The questionnaire consisted of four sections and twenty-five questions: In *Section 1*, background information, personal information (socio-demographic info), self-reported anthropometric measurements (height and weight), and stress level information (DASS-21 based questions); In *Section 2* consisted of physical activity level (weekly exercise duration) while in *Section 3* consisted information of eating behaviors; In *Section 4*, lifestyle behavior (smoking habits, alcohol intake, and sleeping habit) was asked. A copy of the questionnaire can be found in the Extended data. The participants’ answers to the “Accommodation” represent two living conditions of students which are staying at hostel with irregular cooks and living with family with regular cook. While the answers to stress were categorized as either “High Stress” or “Low stress”. As per the DASS-21 stress questionnaires and category, those under severe and extremely severe were reclassified as “High Stress” and those under mild, normal and moderate as “Low Stress”. “Poor Sleep” and “Good Sleep” were determined using the sleeping hours recommendation by National Sleep Foundation for adults aged 18 to 25 years old which is about 7-9 hours. Good sleep is considered for those participants who answered 7 to 9 hours of sleeping. ^18^ Any duration less than 7 hours or more than 9 hours were considered as “poor sleep”. The answers for alcohol drinking were classified into 2 groups, non-drinker which is no alcohol taken for past 30 days and drinker if they have taken alcohol regardless of number of drinks for past 30 days. This is the same for smoking, non-smoker for no cigar or vape for the past 30 days and smoker, if they have taken nicotine regardless of number for past 30 days. Physical Activity in this study less than 150 minutes of moderate-intensity or 75 minutes of vigorous-intensity physical activity is classified as inadequate physical activity, while more than 150 minutes of moderate-intensity or 75 minutes of vigorous-intensity physical activity is classified as adequate physical activity. ^19^ Eating behaviour is assessed by determining the eating habits of the students namely, number of meals per day (<3 meals, 3 meals, >3 meals), servings of fruit and vegetable in a day (1-5, >5 servings), type of diet practiced (vegetarian, non-vegetarian, mixed diet), number of fast foods per week , number of days of breakfast taken in a month Responses are further classified under yes or no for skipping breakfast for the past 30 days; Inadequate or Adequate for daily servings of fruits and vegetables; Regular or Irregular for number of daily meals; rarely or often for consumption of snacks, carbonated soft drinks and fast food; and unvaried or varied diet for type of diet practiced.

A pilot test was conducted on students from a class to determine the questionnaire acceptability and ease of use by the participants and the data collected for analysis. All the suggestions and feedback were collected and integrated accordingly into the questionnaires.

### Data collection

Baseline height and weight were measured by the researchers. Baseline height was used to calculate BMI of the three follow up periods while the weight for Semesters 2 and 3 were measured by the participants at home. Sections 1-4 of the questionnaire were answered online by the participants through Google form in all the study periods.

### Ethical considerations

The questionnaire used in this research was fully typed in English and was approved by Taylor’s University Human Ethics Committee with reference number HEC 2019/119. The active participation in and completion of the online form were taken as consent, and the ethical committee approved the consent protocols and procedures.

### Data analysis

IBM Statistical Package for Social Sciences (SPSS) version 25 was used to describe and analyze the data collected. For descriptive data, frequency, mean and standard deviation (SD) were used to summarize the data. Generalized Estimating Equations (GEE) were employed to test the association between BMI overtime and sex assigned at birth throughout the cohort study. The independence t-test was also used to test the difference between mean BMI and sex assigned at birth over time. The predicted variables associated with BMI were tested using ANOVA one-way analysis. Any variables with p < 0.250 in the ANOVA test were tested again with GLM multivariate analysis for regression analysis. A 95% of confidence interval was fixed throughout the cohort study, with any obtained p-value lesser than 0.05 is considered significant.

## Results

### Descriptive analysis

A total of 50 (17 males and 32 females) first-year medical students from Taylor’s University School of Medicine were included in this study. The response rate was 82% (50/61). One student withdrew from the study during Semester 2. Characteristics of year one medical students who participated in the study are presented in Table 1.

**Table 1.** Characteristic of Year 1 medical students at Taylor’s University, Selangor, Malaysia (2019).

Characteristic	(N = 49) Number (%)
**Age group**	
≥ 21 years old	45 (91.8)
<21 years old	4 (8.2)
**Ethnicity**	
Malay	6 (12.2)
Chinese	19 (38.8)
Indian	18 (36.7)
Others	6 (12.2)
**Gender**	
Male	17 (34.7)
Female	32 (65.3)
**First-degree family history of obesity**	
Yes	5 (10.2)
No	36 (73.5)
Not sure	8 (16.3)
**Accommodation**	
With Parents/Anyone that cooks regularly	38 (77.6)
Alone/Friends/non-regular cooks	11 (22.4)
**Height, cm** (mean ± SD)	164.04 ± 7.31
**Weight, kg** (mean ± SD)	60.06 ± 12.33
**BMI, kg/m** ^**2**^ (mean ± SD)	22.23 ± 3.89

**Table 2.** BMI Classification of Year 1 medical students (Semester 3).

BMI (kg/m ^2^)	BMI category	(N = 49) Number (%)
**WHO body weight classification according to International population ^a^**		
<18.5	Underweight	8 (16.3)
18.5-24.9	Normal	32 (65.3)
**25.0-29.9**	**Overweight**	**5 (10.2)**
**≥30.0**	**Obese**	**4 (8.2)**
**WHO body weight classification according to Asian population ^a^**		
<18.5	Underweight	8 (16.3)
18.5-22.9	Normal	23 (46.9)
**23.0-27.4**	**Overweight**	**13 (26.5)**
**≥27.5**	**Obese**	**5 (10.2)**

^a^ WHO Expert Consultation. ^6^

*Prevalence of obesity among Taylor’s University year one medical students as they progress towards clinical year*

Compared with the national reported prevalence value of obesity in adults (33.7%), ^8^ the prevalence of obesity among the medical students in this study based on the Asian cut-off value was high at 36.7% ( Table 3).

**Table 3.** BMI Classification of year one medical students as they progress towards clinical year.

WHO body weight classification according to Asian population ^a^				
BMI (kg/m ^2^)	BMI category	Semester 1 (N = 50) Number (%)	Semester 2 (N = 49) Number (%)	Semester 3 (N = 49) Number (%)
< 18.5	Underweight	7 (14.0)	10 (20.4)	8 (16.3)
18.5-22.9	Normal	23 (46.0)	24 (49.0)	23 (46.9)
**23-27.4**	**Overweight**	**11 (22.0)**	**8 (16.3)**	**13 (26.5)**
**≥ 27.5**	**Obese**	**9 (18.0)**	**7 (14.3)**	**5 (10.2)**

^a^ WHO Expert Consultation. ^6^

### Bivariate and multivariate analysis

*Comparison of mean BMI changes of Taylor’s University year one medical student over a year*

**Figure 1.**
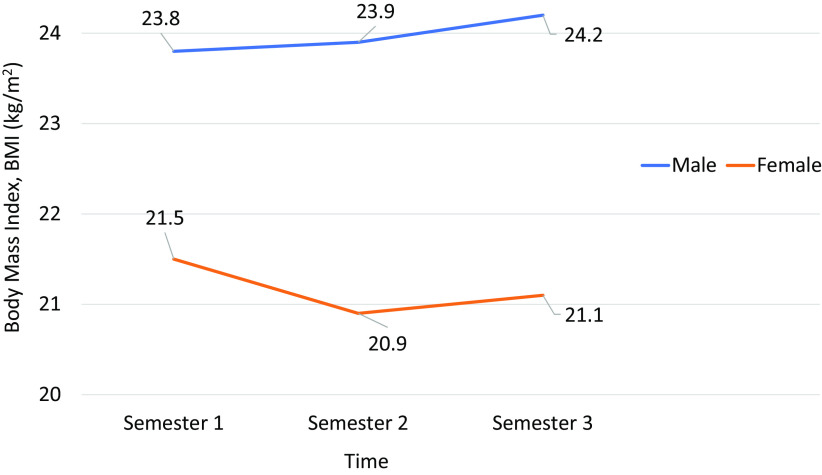
Comparison of mean BMI overtime by gender.

Based on the BMI result obtained ( Table 4), the difference between mean BMI overtime in three studies was found not significant (p > 0.05). On the contrary, the difference between gender and BMI over time in this study was notable (p < 0.05) ( Table 5). As shown in Table 5, there is no difference (p > 0.05) in mean BMI between the gender during Semester 1. However, during Semesters 2 and 3, the BMI among the males was significantly higher (p < 0.05) compared with females (refer to Figure 1).

**Table 4.** Comparison of mean BMI overtime in three periods.

Time	BMI, kg/m ^2^ (Mean ± SD)	p-Value
Semester 1	22.86 ± 0.65	
Semester 2	22.51 ± 0.60	0.091
Semester 3	22.73 ± 0.60	

**Table 5.** Comparison of mean BMI overtime by gender.

Time	Gender	BMI, kg/m ^2^ (Mean ± SD)	p-Value
Semester 1	Male	23.83 ± 4.74	0.058
	Female	21.47 ± 3.63	
Semester 2	Male	23.87 ± 4.70	**0.009**
	Female	20.91 ± 2.88	
Semester 3	Male	24.17 ± 4.33	**0.008**
	Female	21.10 ± 3.29	

*Evaluation of risk factors associated with BMI among Taylor’s University year one medical student*

When the predicted variables were tested with ANOVA one-way analysis, significant associations (p < 0.05) were found between BMI with accommodation and stress level. Other predicted variables were not significantly associated with BMI, but variables (Sleeping habit and Carbonated soft drinks intake) with p < 0.250 were then subjected to GLM Multivariate Analysis.

There were significantly lower average BMI levels of those who can cook/live with people who can cook, 21.53 kg/m ^2^, compared to 24.63 kg/m ^2^ of those who cannot cook/living with people who cannot cook. This is also seen in the p-value of 0.018, indicating great significance between this factor and BMI/weight gain. Based on Table 6, we can assume stress levels are among the leading players in determining the change in a medical student’s BMI. This can be seen in the participants’ mean BMI levels, which are the highest recorded average at 26.57 kg/m ^2^ (p-value = 0.018).

**Table 6.** Factors associated with BMI.

Variables	Mean ± SD	F	p-Value
**Accommodation**			
With Parents/Anyone that cooks regularly	21.53 ± 3.38	6.016	**0.018**
Alone/Friends/non-regular cooks	24.63 ± 4.68		
**Stress level**			
Low	21.84 ± 3.49	5.993	**0.018**
High	26.57 ± 5.98		
**Sleeping habit**			
Poor	23.03 ± 4.59	1.456	0.244
Good	21.10 ± 3.27		
**Carbonated soft drinks intake in the last 30 days**			
Rarely	22.04 ± 3.80	1.435	0.239
Often	23.91 ± 4.80		
**Alcohol consumption**			
Non-drinker	22.24 ± 3.93	0.017	0.897
Drinker	21.72 ± 0.04		
**Cigarette smoking**			
Non-smoker	22.24 ± 3.92	0.017	0.897
Smoker	21.72 ± 0.04		
**Physical activity/week**			
Inadequate	22.06 ± 4.47	0.111	0.741
Adequate	22.44 ± 3.03		
**Number of meals/day**			
Regular	22.30 ± 4.13	0.016	0.901
Irregular	22.16 ± 3.72		
**Adequacy of fruits & vegetable serving/day**			
Inadequate	22.33 ± 3.82	0.022	0.883
Adequate	22.16 ± 4.00		
**Number of snacks/day**			
Rarely	22.19 ± 3.35	0.006	0.938
Often	22.28 ± 4.65		
**Habit of skipping breakfast**			
Yes	22.49 ± 4.05	0.247	0.621
No	21.93 ± 3.77		
**Number of fast foods/week**			
Rarely	21.81± 3.58	1.273	0.265
Often	23.17 ± 4.50		
**Type of diet**			
Unvaried diet	22.96 ± 0.62	0.168	0.846
Varied diet	21.76 ± 4.05		

After GLM Multivariate analysis, it revealed that accommodation (p-value = 0.016), stress level (p-value = 0.021) and sleeping habit (p-value = 0.011) were the independent predictor of BMI changes in year one medical students ( Table 7).

**Table 7.** GLM Multivariate analysis.

Variables	Odds Ratio	95%	CI	p-value
**Accommodation**				
With parents/Anyone that cooks regularly	1.227	-5.214	-0.541	**0.016**
Alone/Friends/Non-regular cooks				
**Stress level**				
Low	1.314	-7.408	-0.592	**0.021**
High				
**Sleeping habit**				
Poor	0.866	0.572	-4.337	**0.011**
Good				
**Carbonated soft drinks intake in the last 30 days**				
Rarely	1.120	-3.848	-2.520	0.683
Often				

## Discussion

The predominant proportion of participants who had both higher BMI and also an increased BMI overtime is the male sex (assigned at birth) (refer to Table 5 and Figure 1). This is consistent with study findings conducted on medical students in Malaysia, ^15^ India, ^11^ and in Pakistan. ^20^ On the contrary, the findings of our study contradicted the findings of two studies conducted by Anupama ^21^ and Fernandez, ^22^ which showed that the predominant proportion of their medical students who have higher BMI overtime is female. All the above mentioned studies were conducted through cross-sectional study design where there is generally no evidence of the temporal relationship between the variables and BMI, thus could not establish causality. Our cohort study shows the temporal relationship between the predicted variables where BMI changes can be identified over time. However, the presence of predominant proportion of male medical students with higher BMI might be due to their higher muscle mass than female (sex assigned at birth) students.

In our study, accommodation turned out to be the most significant factor in determining risk factors for weight gain. This finding is in line with other studies, which also mentioned that people who had more home-cooked meals in their weekly diet are less obese than those who do not. ^23^^–^^25^ Similar studies from a Pakistan medical school also reported that medical students who had more meals at home reported lower obesity rates than those having food outside their home. ^20^ A possible reason could be that foods consumed outside are usually higher in fat-content and also contain unhealthy additions than home-cooked food. Our research shows that medical students who lived alone/or cannot cook themselves had higher BMI values than medical students who could cook/live with people who could cook.

Our study revealed that the number of medical students who experienced high levels of stress has decreased over the year, which might be possibly because most of the participants were living with their parents during this period. This might help lessen the medical students’ burden as they receive the best support-system at their homes. However, a higher BMI value is noticed in medical students with high stress, which might be due to the sudden shift in learning methods where students were forced to adapt to online learning. Similar findings were found from studies done in America which also found that medical students are experiencing severe stress and thought that online learning is a burden. ^26^

Our findings on sleep are similar with Eveline. ^27^ We found that majority (59.2%) of medical students having a poor sleep compared to their 60%. From this, we found medical students who experienced poor sleep had a higher average BMI than those who experienced good sleep, which correlates with research findings from Zailinawati *et al.* ^28^ This might be due to the intense workload and vast topic to learn in the medical field that often leads to a sacrifice in sleeping time for revisions. However, the findings of this study are not supported by the recent study done by Hameed. ^29^

There were some limitations of this study **.** This study was done during the COVID-19 pandemic, though the baseline data was collected few months before the pandemic. BMI was calculated using measured weight and height during Semester 1, while BMI for the subsequent periods were calculated using the baseline height and their self-reported weight. The movement restrictions during follow ups which required all education sectors to shift to online classes might have contributed to the outcome of the study.

## Conclusion

The prevalence of obesity and overweight in Taylor’s University year one medical students was considered high in all three semesters (40%, 30.6% and 36.7%). A year of follow-up suggests that there is an increase in the BMI of male students and the changes are associated with changes in accommodation, stress levels, and sleeping habits.

### Recommendations

There must be a radical change in medical education. Medical students should be given a balanced lifestyle, with time for adequate sleep, and exercise, and access to a proper diet. Online studies which are becoming more popular even before the pandemic should be combined with some hybrid form of learning to increase the physical activity of students.

## Data Availability

Harvard Dataverse: OBESOGENIC ENVIRONMENT IN THE MEDICAL FIELD: FIRST YEAR FINDINGS FROM A 5-YEAR COHORT STUDY, https://doi.org/10.7910/DVN/7G1VD8. ^30^ This project contains the following underlying data:
• SPSS Merged File (Repeated Measures) Harvard Dataverse: OBESOGENIC ENVIRONMENT IN THE MEDICAL FIELD: FIRST YEAR FINDINGS FROM A 5-YEAR COHORT STUDY, https://doi.org/10.7910/DVN/7G1VD8. ^30^ This project contains the following extended data:
• A copy of the questionnaire Data are available under the terms of the Creative Commons Zero “No rights reserved” data waiver (CC0 1.0 Public domain dedication).

## References

[1] Powell P Spears K Rebori M : *What is obesogenic environment?* University of Nevada;2010 [cited 20 July 2021]. Reference Source

[2] Swinburn B Egger G Raza F : *Dissecting obesogenic environments: the development and application of a framework for identifying and prioritizing environmental interventions for obesity.* Vol.29. ELSEVIER;1999; pp.563–570. 10.1006/pmed.1999.0585 10600438

[3] Obesity and overweight: World Health Organization, Int. c2020 [20 July 2021]. Reference Source

[4] Prevalence of obesity: World Obesity Federation. c2019 [20 July 2021]. Reference Source

[5] Reference Source

[6] WHO Expert Consultation (WHO) : Appropriate body-mass index for Asian population and its implications for policy and intervention strategies. *Lancet.* 2004;10:157–163. 10.1016/S0140-6736(03)15268-3 14726171

[7] Institute for Public Health (IPH) 2008: *The third national health and morbidity survey (NHMS III).* Kuala Lumpur: Ministry of Health Malaysia;2006.

[8] Institute for Public Health (IPD): *National health and morbidity survey 2019 (NHMS 2019). Vol. I: NCDs – Non-communicable Diseases: Risk Factors and other Health Problems.* Kuala Lumpur: Ministry of Health Malaysia;2019.

[9] *Malaysia ‘most obese Asian country’.* BBC News, Int.;c2020 [20 July 2021]. Reference Source

[10] Barnett K : Physician obesity: the tipping point. *Glob. Adv. Health Med.* 2014;3(6):8–10. 10.7453/gahmj.2014.061 PMC4268640 25568827

[11] Thomas E Geethadevi M : Prevalence and determinants of overweight and obesity among medical students. *Natl. J. Physiol. Pharm. Pharmacol.* 2019;01:1–7. 10.5455/njppp.2020.10.1035506112019

[12] Bertsias G Mammas I Linardakis M : Overweight and obesity in relation to cardiovascular disease risk factors among medical students in Crete, Greece. *BMC Public Health.* 2003;3:3. 10.1186/1471-2458-3-3 12517305 PMC140012

[13] Bleich S Bennett W Gudzune K : Impact of physician BMI on obesity care and beliefs. *Obesity.* 2012;20(5):999–1005. 10.1038/oby.2011.402 22262162 PMC3645927

[14] Abramson S Stein J Schaufele M : *Personal exercise habits and counseling practice of primary care physicians: a national survey.* Lippincott Williams & Wilkins Inc.;2000 [20 July 2021]; vol.10:40–48. Reference Source 10.1097/00042752-200001000-00008 10695849

[15] Boo NY Chia GJ Wong LC : The prevalence of obesity among clinical students in a Malaysian medical school. *Singap. Med. J.* 2010;51(2):126–132. 20358151

[16] Galvan JAA Sriram S Chinna K : Low prevalence of Overweight and obesity among medical students at a University in Malaysia. *Southeast Asian J. Trop. Med. Public Health.* 2019;50(6):1179–1187.

[17] Alcohol and public health: Centers for Disease Control and Prevention. c2020 [20 July 2021]. Reference Source

[18] Paruthi S Brooks LJ D’Ambrosio C : Recommended Amount of Sleep for Pediatric Populations: A ConsensusStatement of the American Academy of Sleep Medicine. *J. Clin. Sleep Med.* 2016 Jun15;12(6):785–786. 27250809 10.5664/jcsm.5866 PMC4877308

[19] Ministry of Health Malaysia: *Clinical practice guidelines on primary & secondary prevention of cardiovascular disease.* Kuala Lumpur: Ministry of Health Malaysia;2017.

[20] Mahmood S Perveen T Najjad M : Overweight and obesity among medical students of public sector’s institutes in karachi, Pakistan. 2013 [cited 20 July 2021]. Reference Source

[21] Anupama M Iyengar K Rajesh S : A study on prevalence of obesity and life-style behavior among medical students. *Research Gate.* 2017;4:3314–3317. 10.18203/2394-6040.ijcmph20173836

[22] Fernandez K Singru S Kshirsagar M : Study regarding overweight/obesity among medical students of a teaching hospital in pune, India. *Med. J. DY. Patil. Univ.* 2014;7:279–283. 10.4103/0975-2870.128950

[23] Tani Y Fujiwara T Doi S : Home Cooking and Child Obesity in Japan: Results from the A-CHILD Study. 2019 [cited 20 July 2021]. Reference Source Reference Source 10.3390/nu11122859 PMC6950631 31766554

[24] Susanna M Heather B Wendy W : Frequency of eating home cooked meals and potential benefits for diet and health: cross-sectional analysis of a population-based cohort study. *BMC.* 2017 [cited 20 July 2021];14:109. 10.1186/s12966-017-0567-y 28818089 PMC5561571

[25] David C Kylie B Gita M : *Which food-related behaviours are associated with healthier intakes of fruits and vegetables among women?* Cambridge University Press;2007 [cited 20 July 2021]. Reference Source 10.1017/S1368980007246798 17288623

[26] Eric W : American Medical Student Perceptions of the Online Learning Environment, their Quality of Life, and the School of Medicine’s Response During COVID-19 Pandemic. [cited 20 July 2021]. Reference Source Reference Source

[27] Evelina P Darius L Virginija A : Associations of quality of sleep with lifestyle factors and profile of studies among Lithuanian students MDPI. 2010 [cited 20 July 2021]. Reference Source 20966622

[28] Zailinawati AH Teng CL Chung YC : Daytime sleepiness and sleep quality among Malaysian medical students. 2009 [cited 20 July 2021]. Reference Source Reference Source 20058567

[29] Hameed R Bhat A Nowreen N : Prevalence of overweight and obesity among medical students and its correlation with sleep pattern and duration. *ResearchGate.* 2019;6. 10.21276/ijcmr.2019.6.6.6

[30] Galvan JA : OBESOGENIC ENVIRONMENT IN THE MEDICAL FIELD: FIRST YEAR FINDINGS FROM A 5-YEAR COHORT STUDY[Dataset]. *Harvard Dataverse.* 2021. 10.7910/DVN/7G1VD8

