# Supplementary material for: Obesogenic Environment in the medical field: First year findings from a five-year cohort study
Source: F1000Res. 2023 May 25;12:550. [Version 1] doi: 10.12688/f1000research.125203.1 (PMC10589624; doi:10.12688/f1000research.125203.1)
Supplement: Peer review history

# Reviewer report: Reviewer response for version 1

**DOI:** 10.5256/f1000research.137483.r209970

**Published:** 2023-10-20

**Authors:** Selvamani Y

**Affiliations:** 1 School of Public Health, SRM Institute of Science and Technology, Chennai, Tamil Nadu, India

This is an interesting article about obesogenic environment in the medical field. The rates of obesity are increasing in the general population. Medical students, as future physicians, have an important role and responsibility to play in advising our society about obesity and also treating obesity and the related wide spectrum of metabolic illnesses that accompany obesity. This article is particularly very interesting and useful because the study highlights the contribution and effects of the living environment of medical students to the changing patterns of BMI. This is particularly important in the scenario with epidemiological burden and transition with increasing incidence of non-communicable diseases even affecting the younger generations. The results suggest regular monitoring of the physical health of the students is important. The implications of this study could be used for wider policy implications and bringing about changing and lifestyle modification interventions in the medical field among both the medical and allied health science students. I accept the paper without any reservations considering the importance of the issue addressed in the paper and the robust methodology used here. Given the cohort nature of this study, further research that is done in this area will help to establish causality which will serve very vital for policy development in the area. It is necessary to be sure that medical students are in good health and without obesity before they can take up the advisory and treating role as a physician in the community.

 I believe that the paper is good in its current format and I accept it. I would like to advise that making the following changes would be good. Table 7 adjusted for other covariates such as socio-demographic variables.  It would be better if Table 1 had all other covariates also.

Is the work clearly and accurately presented and does it cite the current literature?

Yes

If applicable, is the statistical analysis and its interpretation appropriate?

Yes

Are all the source data underlying the results available to ensure full reproducibility?

Yes

Is the study design appropriate and is the work technically sound?

Yes

Are the conclusions drawn adequately supported by the results?

Yes

Are sufficient details of methods and analysis provided to allow replication by others?

Yes

Reviewer Expertise:

Demography, Social epidemiology and Food and nutrition security

I confirm that I have read this submission and believe that I have an appropriate level of expertise to confirm that it is of an acceptable scientific standard.

# Reviewer report: Reviewer response for version 1

**DOI:** 10.5256/f1000research.137483.r204772

**Published:** 2023-09-25

**Authors:** Gajanan Dattatray Velhal

**Affiliations:** 1 Community Medicine, Seth G S Medical College and KEM Hospital, Mumbai, Maharashtra, India

Present cohort study, in progress, undertaken by Jo Ann Andoy-Gavlan et al, at Malaysia need special appreciation for having touched the important aspects in the life of Medical students during their study period in the medical college. It is well known that getting into the environment of Medical College after completing conventional education in schools/junior college, invites lot of adjustments/modifications in the behaviour patterns of the students, that too at their tender stage in life i.e. late adolescent’s. Contrary to the expectations - in relation to burden of medical studies, stress of getting adjusted to surrounding environment, and being away from parental home, here it is found that there is tendency on the part of the student’s to gain weight and increase BMI, that too more significantly among males as compared to females, in the first year of study . This needs more elaboration by interviewing the participants directly as well as organizing focus group discussions on the topic among students, teachers, care takers in the hostel etc. over and above, collecting the information on questionnaires. Of course the cohort is going to be followed for next  four years, which will provide still more meaningful conclusions to the policy makers and administrator’s to look into this important aspects of medical students with due attention to promote health of students in long run.

Is the work clearly and accurately presented and does it cite the current literature?

Yes

If applicable, is the statistical analysis and its interpretation appropriate?

Yes

Are all the source data underlying the results available to ensure full reproducibility?

Yes

Is the study design appropriate and is the work technically sound?

Yes

Are the conclusions drawn adequately supported by the results?

Yes

Are sufficient details of methods and analysis provided to allow replication by others?

Yes

Reviewer Expertise:

Epidemiology, Research Methodology, Health Planning & Management, MH care, communicable and non communicable diseases

I confirm that I have read this submission and believe that I have an appropriate level of expertise to confirm that it is of an acceptable scientific standard.
